# Editorial: Digital imaging of plants

**DOI:** 10.3389/fpls.2022.986584

**Published:** 2022-08-25

**Authors:** Michele Pisante, Angelica Galieni, Fabio Stagnari, Kathy Steppe, Qingguo Xie, Nicola D'Ascenzo

**Affiliations:** ^1^Faculty of Bioscience and Technology for Food Agriculture and Environment, University of Teramo, Teramo, Italy; ^2^Research Centre for Vegetable and Ornamental Crops, Council for Agricultural and Economics Research (CREA), Pontecagnano Faiano, Italy; ^3^Laboratory of Plant Ecology, Department of Plants and Crops, Faculty of Bioscience Engineering, Ghent University, Ghent, Belgium; ^4^Department of Biomedical Engineering, Huazhong University of Science and Technology, Wuhan, China; ^5^Department of Medical Physics and Engineering, Istituto Neurologico Mediterraneo Neuromed I.R.C.C.S., Pozzilli, Italy; ^6^Department of Electronic Engineering and Information Science, University of Science and Technology of China, Hefei, China

**Keywords:** digital plant imaging, plant positron emission tomography, plant RGB imaging, plant functional imaging, early stress detection

## Digital plant imaging – A revolution on the crossroads between physics, mathematics, plant physiology, and digital agronomy

Each plant species is different and physiological damages following stress are strongly crop-dependent. Visual inspection of morphologic, organoleptic, and chromatic traits of plants has represented the traditional approach to stress assessment since centuries. When addressed from an information science perspective, visual inspection deals with the extraction of parameters of agronomic interest from crop digital images. The arrival of digital imaging and Artificial Intelligence (AI) boosted digital image processing to an unprecedented level of precision, enabling the possibility of identifying hidden features in macroscopic crop images.

The predictive power of these digital imaging techniques is limited by the empiric nature of the data, which relies on external evidence of stress. It is however well-known that plants, when subjected to both biotic and abiotic stressful conditions, respond through physiological and metabolic changes mediated by pulses of gene expression, suggesting the existence of a complex signaling network that allows plant recognizing adverse environmental conditions as well as changes in growth conditions. Modern nuclear imaging techniques, such as Computed Tomography (CT) and Magnetic Resonance Imaging (MRI) allow to visualize in 3D the internal structure of the vascular system and organs of plants with a spatial resolution of few hundred of micrometers and help with the identification of structural evidence of stress.

Also supported by AI classification algorithms, morphologic mesoscopic imaging represents a clear advance with respect to macroscopic digital imaging but does not allow to observe the physiological aspects of plant stress. Positron Emission Tomography (PET) introduced in this landscape a revolution. It is in fact the only technique which allows to visualize in 3D the metabolism and transport of carbon, glucose, and water from sources to sinks, enabling an *in-vivo* and real-time inspection of the functional aspects of plants vascular system and organs. If the development of a plant PET system requires a biomedical and electronic engineering background, the quantitative interpretation of PET measurements requires the development of applied mathematics and physical modeling theories and methods, to form a 3D image and to connect the observation with quantitative fluid dynamic aspects. This unique cross-disciplinary approach is at the basis of a series of European actions, like the Horizon 2020 project PETAL (Positron Emission Tomography for Agriculture and Life) which aims at collecting the first worldwide digital biobank of plant functional imaging data for wheat stress assessment (https://petalproject.eu).

The 17 original research articles, the two methods and two review papers of this Research Topic describe this transition and revolution from conventional agronomic methods to disruptive cross-disciplinary, technological, and AI-supported digital imaging techniques for the early stress assessment on the plant models and crops in green houses and open field.

## The frontier of digital plant imaging—From RGB imaging to dynamic functional imaging

The potential of Red Green Blue (RGB) digital imaging is nowadays widely demonstrated in agronomy. For instance, contour-based detection of fungal stromata has been proven by Lee et al. to be helpful in quantifying the intensity of tar spot infections of corn leaves. Furthermore, visual disease estimation of wheat spike blast can be used to train deep convolutional neural networks (CNN) for disease severity (DS) classification. Results shown by Fernández-Campos et al. provide a promising approach to classify images into three wheat blast severity categories. Along a similar research direction, Zheng et al. detected green citrus in natural environments and Wu et al. offered a solution to the real-time operating requirements for the banana bud-cutting robot. Furthermore, Ravindran et al. tested a computer vision wood identification model for Peruvian woods that is ready for immediate in-country field evaluation on the XyloTron platform.

The high-throughput phenotyping approach (Thoday-Kennedy et al.) allowed to identify candidate genotypes for salt tolerance in safflower. Similarly, Sorrentino et al. used it as a valuable tool to compare biostimulants at different concentrations on plants grown under several conditions, allowing to accelerate the selection of the best performing substances at highly efficiency. A digital plant imaging screening method of novel and effective biological control agents (BCAs) against soil-borne plant pathogens is proposed by Manganiello et al.. Finally, nutrient diagnosis in Menthol mint to make precise N fertilizer recommendations has been introduced by Pandey et al..

Digital plant imaging is going to play a key role in the early assessment of biotic stress, steering fast diagnosis of diseases and timely control measures, which are crucial to increase plant production. Deng et al. carried out an accurate diagnosis on the vegetative part of the rice in open field, and developed a model for plant disease recognition. A similar approach based on deep learning method on multi-scale dense classification network allowed to distinguish different kind of diseases and the same disease with different grades (Tian et al.). Similarly, Fuentes et al. propose a paradigm called “control to target classes.” The core of this approach is to train and validate a deep learning-based detector using target and control classes on images collected in various greenhouses. On the same topic, Gu et al. proposed an improved method for the diagnosis of hot-pepper diseases and pests using a fine-tuning-based transfer learning method.

As mentioned above, macroscopic digital imaging allows to extract features, which can be correlated to a specific state of the plant. However, to produce a quantitative biomarker containing physiological information, mesoscopic and microscopic imaging will be necessary. The review (Payne and Kurouski) on Raman spectroscopy (RS) discusses the most recent research articles on this emerging analytical technique that can be used for non-invasive, non-destructive, and confirmatory diagnostics of diseases, as well as the nutrient deficiencies in plants. The evaluation of herbicide resistance and stress response in plants under field conditions with RS is presented by Singh et al. as well as the attempt by Payne et al. for the identification of peanut genotypes and nematode resistance.

The cross-fertilization between agronomy and nuclear imaging techniques has been beneficial to extend the imaging potential of existing instrumentation. For instance, Krzyzaniak et al. have demonstrated that neutron tomography is relevant to quantify the root volume in grapevine propagated by cuttings potentially be extended to other plants. As mentioned above, nuclear imaging techniques represent the frontier of plant imaging. Among the several existing technologies, Positron Emission Tomography (PET) represents the only solution for non-invasive, quantitative, dynamic functional imaging. PET enables agronomists and plant scientists to visualize the 3-dimensional flow of nutrients within the plant vascular system and the organs, identifying cellular metabolism sources and storage sinks (Galieni et al.). Mincke et al. highlighted in their guide to plant-PET that this type of imaging is a complex cross-disciplinary topic, requiring a background in biomedical engineering, mathematical modeling, plant science, applied and nuclear physics, which are at the basis of a proper implementation and use of the PET technique on plants. Such a cross-disciplinarity represents at once the difficulty and the scientific challenge of plant PET, motivating the development of novel dedicated plant imaging systems (Antonecchia et al.). Plant PET has been found successful in the quantitative study of plant metabolism. For instance, Mincke et al. developed a technique for the study of carbon dynamics based on [11C]-CO_2_. Similarly, Miyoshi et al. studied carbon metabolism and transport in strawberry fruits using the PETIS system, developed for the specific application of plant-PET.

Plant digital imaging represents one of the key emerging technologies for the ecological intensification as a pathway to sustainable agriculture through accurate agronomic information. We expect that it will provide an effective beneficial result in the early detection and control of crop stress. This field of research became fundamental due to the rapidly devastating conditions caused by global climate change and desertification, also exacerbated by the recent economical and societal developments.

## Author contributions

ND'A and MP wrote the manuscript. AG, FS, KS, and QX read the manuscript and implemented corrections. All authors contributed to the article and approved the submitted version.

## Conflict of interest

The authors declare that the research was conducted in the absence of any commercial or financial relationships that could be construed as a potential conflict of interest.

## Publisher's note

All claims expressed in this article are solely those of the authors and do not necessarily represent those of their affiliated organizations, or those of the publisher, the editors and the reviewers. Any product that may be evaluated in this article, or claim that may be made by its manufacturer, is not guaranteed or endorsed by the publisher.

